# The Effect of Hormone Replacement Therapy on Dry Eye Syndrome Evaluated with Schirmer Test and Break-Up Time

**DOI:** 10.1155/2015/420302

**Published:** 2015-11-18

**Authors:** Yanhong Feng, Gang Feng, Shuli Peng, Hui Li

**Affiliations:** ^1^First Affiliated Hospital of Liaoning Medical University, Jinzhou, Liaoning 121000, China; ^2^Department of Ophthalmology, Huludao Central Hospital, Huludao, Liaoning 121000, China; ^3^Department of Ophthalmology, Shanghai Jiao Tong University Affiliated Sixth People's Hospital, Shanghai 200233, China

## Abstract

Hormone replacement therapy (HRT) for dry eye syndrome (DES) is controversial in clinical practice. The goal of this study was to review relevant studies and analyze the pooled data to determine whether HRT is effective for DES. In this study, a literature search of PubMed, Embase, and Cochrane databases up to May 2015 was performed, with the search restricted to English language publications. The studies were screened after reading the abstract and full text. Only studies related to the effect of HRT on DES were included in the meta-analysis. Results of Schirmer tests with and without anesthetics and tear break-up time (BUT) values data were extracted and entered into RevMan software to meta-analyze the overall effect of HRT on DES. A total of 43 studies were identified, and 21 of these studies were found to be related to the effect of HRT on DES. Ultimately, 5 studies were included in the final meta-analysis. The pooled results revealed that HRT can affect Schirmer test results without anesthetics but does not affect Schirmer test results with anesthetics and BUT. The results indicate that HRT might improve DES symptoms when measuring basal tear production without anesthesia.

## 1. Introduction

Dry eye syndrome (DES) is a multifactorial disorder of the tears and ocular surface that results in symptoms of discomfort, visual disturbance, and tear film instability with potential damage to the ocular surface [1]. Typical symptoms include dryness, irritation, itching, fatigue, foreign body sensation, pain, burning, and sensitivity to light in eyes. More severe cases may present as eyes swelling, redness, corneal epithelium damage, and even vision disturbance [1, 2]. DES is common in population with a high prevalence that up to 25% of patients in ophthalmic clinics have symptoms of DES [3, 4].

The main mechanism of DES includes aqueous tear deficiency, excessive evaporation, and inflammation [1]. To date, the most popular treatment for DES patients is the application of artificial tear supplements. However, this therapy is reported to be only palliative, as it only remedies tears deficiency and lubricates ocular surface but not focusing on the pathophysiological fundamentals. Meanwhile, DES symptoms are very common in postmenopausal women and the presence of hormone receptors in eye surface suggested that gonadal steroid hormones may play an important role in regulating tear film function [5]. This was supported by the studies that the tear production and stability were reduced in postmenopausal women, and hormone replacement therapy (HRT) could recover Schirmer test values to within the normal range [6, 7]. However contradictory results also exist. Some studies reported a greater DES incidence in women on HRT compared to those not undergoing the treatment [8, 9]. These conflicting results demonstrate that the effects of systemic hormone therapy on postmenopausal women with DES are under debate. The different results may come from differences in the HRT administration schemes, drug type, drug dose, patient inclusion criteria, severity of DES, follow-up time, and so on. Therefore, it is necessary to review related studies and to pool the different results to clearly elucidate the effects of HRT on DES patients. To the best of our knowledge, this study is the first meta-analysis of the effect of HRT on DES.

## 2. Materials and Methods

Our meta-analysis was conducted in strict accordance with the Preferred Reporting Items for Systematic Reviews and Meta-Analyses (PRISMA) statement, which is a reporting guideline for meta-analyses [10]. The search was performed in the PubMed, Embase, and Cochrane databases up to March 2015. The search terms were “dry eye” OR “dry eye syndrome” AND “hormone replacement therapy” OR “estrogen” OR “sex hormone”. The language of publication was limited to English. Additionally, we manually searched for relevant published studies and review articles.

In the current meta-analysis, we evaluated only studies that (1) assessed postmenopausal women at least one year; (2) obtained consent from the patients; (3) included DES patients who had undergone HRT with any route except topical application; (4) had data obtained from control patients who were given placebo or no HRT treatment; and (5) included Schirmer test values before and after HRT.

Two investigators (Hui Li and Gang Feng) independently screened all identified studies using the above-mentioned criteria. When any disagreement emerged, a third reviewer (Yanhong Feng) participated in the resolution of the issue by discussion.

### 2.1. Quality Assessment

The quality of each study was independently assessed with Jadad scores by two investigators (Hui Li and Yanhong Feng). The Jadad score rates quality according to the following aspects: randomization generation, allocation concealment, and blinding and withdrawal reporting. Scores of 1–3 points are considered low quality, while scores of 4–7 points are considered high quality. The Jadad scores of the included studies are shown in Table 1.

### 2.2. Meta-Analysis

Meta-analyses were then conducted to assess the changes in Schirmer test scores in mm (i.e., the difference in Schirmer test scores before the beginning the study and after treatment) and the change in tear break-up time (BUT; i.e., the difference in BUT values before beginning the study and after treatment). Mean differences (MDs) and 95% confidence intervals (CIs) were estimated for changes in Schirmer test scores and BUT. The Cochrane *I* ^2^ index was calculated to assess heterogeneity, and if the data were not significant (*I* ^2^ < 40%), the MDs were pooled according to a fixed-effect model. Otherwise, a random-effect model was used. The statistical significances of the pooled MDs were evaluated using the *Z*-test. Possible publication bias was assessed with funnel plots. The meta-analyses were performed using the Review Manager (RevMan, version 5.2) from the Cochrane Collaboration [11, 12].

## 3. Results

### 3.1. Search Results

**Figure 1:**
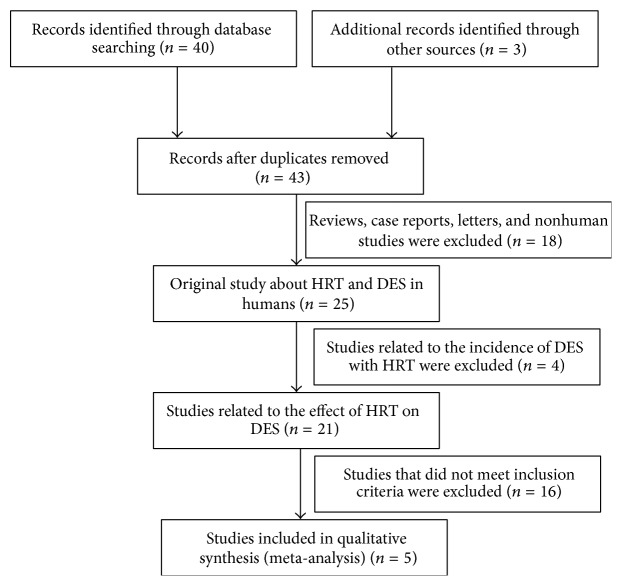
Flow diagram of the study identification, eligibility, and inclusion process.

The articles used were initially identified by electronic and manual searching. After a review of the titles and full texts, we excluded reviews, case reports, letters, and nonhuman studies. The remaining 25 articles were divided into two categories: 21 studies were related to the effect of HRT on DES in postmenopausal women, while 4 studies were related to the incidence of DES in postmenopausal women treated with HRT. As the aim of this meta-analysis is to review whether HRT could improve the symptoms of DES, the 4 articles belonging to the second category were excluded. Among the 21 studies belonging to the first category, 16 studies that did not meet the inclusion criteria were excluded. The reasons for exclusion are as follows: 8 studies did not have control groups; the control groups in 4 studies were given some treatments; 3 studies did not perform Schirmer test; and 1 study used topical hormone. Finally, 5 studies that met the inclusion criteria were included in the meta-analysis. The flow chart of the articles selection process is shown in Figure 1.

### 3.2. Study Characteristics

The inclusion criteria for DES in all the eligible studies were similar. The general characteristics of the included studies and the details of HRT administration are summarized in Table 1. Among the five included studies, three studies [6, 7, 13] were random controlled trials (RCTs) and two studies were not [14, 15]. The Jadad scores for the three RCTs were 3 [6], 4 [7], and 6 [13], respectively (see Table 1).

The detailed characteristics of the subjects in the included studies are summarized in Table 2. One study only reported the age range of the subjects but did not report mean age and standard deviation [13]. Two studies did not report detailed menopause duration but only described it as at least 1 year [6, 13].

The Schirmer test scores and BUT values before beginning the study and after treatment and the calculated difference in Schirmer test and BUT values are summarized in Tables 3 and 4, respectively. The formula for calculating the difference was as follows [16]:

(1) Meandifference=Meanafter treatment−Mean before beginning of the study,SD2diff=SD2after+SD2before−2R∗SD2after∗SD2before R=0.5,SDdiff=SDdifference;SDafter=SDafter treatment;SDbefore=SDbefore beginning of the study.

During data extraction, all five included studies performed a Schirmer test. However, the Schirmer test results before treatment did not have the same baseline. This may be related to the method of preforming the Schirmer test. One study indicated that the Schirmer test was performed under anesthesia [14], while one study performed the test without anesthetics [6]. One study indicated that the Schirmer test was performed with and without anesthetics [7], whereas the other two studies did not mention whether the Schirmer test was performed under anesthesia [13, 15]. Although the final input for the meta-analysis was the difference value but not the exact data obtained before and after treatment, this was influenced by the method used to obtain the Schirmer test results. Therefore, we analyzed Schirmer test results with and without anesthetics separately. Actually, in routine clinical practice, standard definitions of the Schirmer test are based on the amount of wetting measured in 5 min without anesthesia [17, 18]. Thus, Schirmer test results obtained from two studies in which anesthesia was not mentioned were considered as results obtained without anesthetics and were pooled with those obtained without anesthetics.

Additionally, Schirmer test was measured at 3- and 6-month follow-up periods in Affinito's study [7], but only data at 6 months were extracted and input for final analysis. Conversely, the Schirmer test and BUT results obtained from reversed groups [13] (i.e., each group had undergone HRT and control treatments and a washout period was given between the two treatments) in Scuderi's study were extracted as independent data and input for final analysis. BUT results were obtained from three studies with similar methods.

### 3.3. Sensitivity Analysis

To evaluate the sensitivity of the meta-analysis, each study was sequentially excluded from the meta-analysis, and the corresponding heterogeneity results and results of the tests for overall effect are shown in Table 5. As shown in Table 5, the heterogeneities of Schirmer score change with anesthetics could not be obtained after one study was excluded, because only two studies were included in the quantitative analysis. Moreover, the overall effect for these two indexes switched between significant and nonsignificant when any one study was excluded from the meta-analysis. The heterogeneities and overall effect of Schirmer score change without anesthetics and BUT change did not alter significantly when excluding any study from the meta-analysis, with the heterogeneity of Schirmer score change without anesthetics changing between 74% and 98% and all* P* values of overall effect remaining less than 0.05 and heterogeneity of BUT change varying between 98% and 99% and a* P* value of overall effect greater than 0.05.

### 3.4. Meta-Analysis Results of Schirmer Score Change with Anesthesia

**Figure 2:**
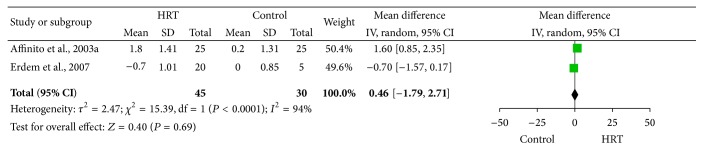
Forest plot of Schirmer score change with anesthetics. Study of Affinito et al. (2003a) indicates that the data were obtained with anesthetics. HRT: hormone replacement therapy; SD: standard deviation; CI: confidence interval; IV: inverse variance.

The data obtained with and without anesthesia were analyzed separately. The data for meta-analysis of Schirmer score change with anesthesia were derived from two studies of 45 cases in the HRT group and 30 cases in the control group. As shown in Figure 2, the heterogeneity of the Schirmer score change was statistically significant (*I* ^2^ = 94%), and thus a random-effect model was used. The MD and 95% CI of the changes in Schirmer test results were 0.46 and −1.79 to 2.71, respectively. The test for the overall effect revealed that the effect of HRT on the Schirmer score change with anesthetic in DES patients was not statistically significant (*Z* = 0.40, *P* = 0.69).

### 3.5. Meta-Analysis Results of Schirmer Score Change without Anesthesia

**Figure 3:**
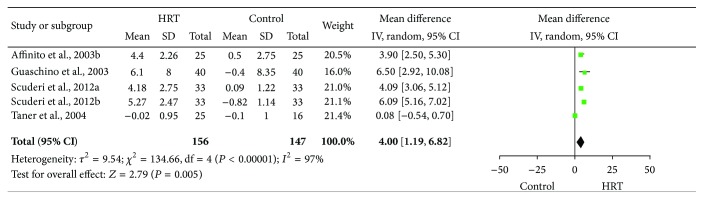
Forest plot of Schirmer score change without anesthetics for the effect of HRT on DES patients. Study of Affinito et al. (2003b) indicates that the data were obtained without anesthetics. Study of Scuderi et al. (2012a) indicates that the data were extracted with the HRT group and control group, and study of Scuderi et al. (2012b) indicates that the data were extracted from reversed groups; that is, the HRT group and control group were reversed after a washout period. HRT: hormone replacement therapy; SD: standard deviation; CI: confidence interval; and IV: inverse variance.

The data for meta-analysis of Schirmer score change without anesthesia were derived from four studies (five data sets) of 156 cases in the HRT group and 147 cases in the control group. As shown in Figure 3, the heterogeneity of the Schirmer score change was statistically significant (*I* ^2^ = 97%), and thus a random-effect model was used. The MD and 95% CI of the changes in Schirmer test scores were 4.00 and 1.19–6.82, respectively. The test for the overall effect revealed that the effect of HRT on the Schirmer score change without anesthetics in DES patients was statistically significant (*Z* = 2.79, *P* = 0.005).

### 3.6. Meta-Analysis Results of BUT Change

The meta-analysis data were derived from three studies (four data sets) of 111 cases in the HRT group and 87 cases in the control group.

**Figure 4:**
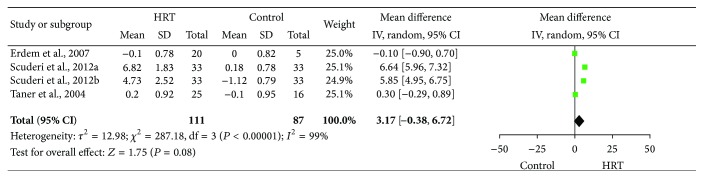
Forest plot of BUT changes using a random-effect model for the effect of HRT on DES patients. BUT: break-up time; HRT: hormone replacement therapy; DES: dry eye syndrome; SD: standard deviation; CI: confidence interval; and IV: inverse variance.

As shown in Figure 4, the heterogeneity of the BUT change was statistically significant (*I* ^2^ = 99%), and thus a random-effect model was used. The MD and 95% CI of BUT change were 3.17 and −0.38 to 6.72, respectively. The test for the overall effect revealed that there was no statistically significant effect of HRT on DES patients (*Z* = 1.75, *P* = 0.08).

### 3.7. Publication Bias

**Figure 5:**
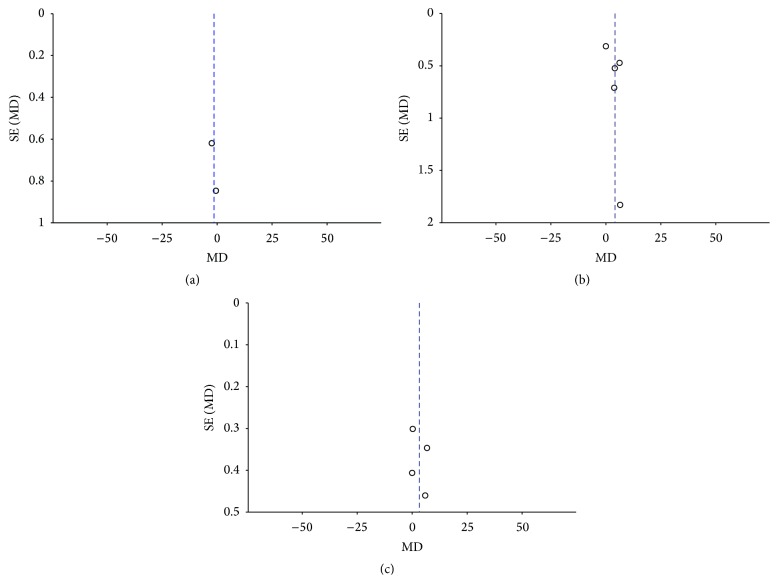
Funnel plot of the change in Schirmer test with (a) and without (b) anesthetics and break-up time (c).

Potential publication bias in the meta-analysis was assessed by funnel plot. As shown in Figure 5, the funnel plot exhibited symmetry, which indicates that there is a lack of publication bias in the results of the meta-analysis.

## 4. Discussion

The results of the present meta-analysis showed that, compared with control treatment, HRT can increase Schirmer test scores without anesthesia but does not increase Schirmer test scores with anesthesia. Schirmer test can be performed with and without topical anesthesia. Schirmer test without anesthesia measures reflective tear secretion, whereas it measures basal lacrimal secretion under topic anesthesia [19, 20]. Schirmer scores with anesthesia were lower than those without anesthesia [21, 22]. Our results suggest that HRT can affect reflective tearing more than basal tearing.

BUT meta-analysis indicated that HRT could not improve the tear stability in DES patients. This is the same as the results for Schirmer scores obtained with anesthetics but is contrary to those obtained without anesthetics. BUT is defined as the time between the first black dot and the last blink when opening eyes with horizontal sight and it represents tear film stability on the ocular surface [23]. The different aspects of tear production measured by Schirmer test and BUT, together with the complex meta-analysis results, indicate that HRT can improve the quantity of tears without anesthetics, but it cannot improve the stability of tears in DES patients.

Potential limitations to this meta-analysis should be considered. First, not all the included studies are RCTs. In fact, there are only three RCTs, and two non-RCTs were included in the final analysis. Second, the sample sizes of the included studies are small. Third, DES severity was not evaluated in all included studies, which may affect the outcome of HRT on DES. DES severity is typically calculated, by the Delphi and International Dry Eye WorkShop panels, on a scale of 1 to 4, with the latter being the most severe [24]. The outcomes of treatment are closely related to disease severity. Therefore, further RCT studies that include subgroups of DES patients according to disease severity are needed in the future.

## 5. Conclusions

To the best of our knowledge, this meta-analysis is the first to summarize data regarding DES patients who have undergone HRT. Based on the data available in the literature, HRT is capable of significantly improving tear production as indicated by the increased reflex tearing evaluated by Schirmer test without anesthesia. Given that most cases of DES were not further grouped according to disease severity in the included studies, the overall effect of HRT on DES patients should be interpreted carefully. Future RCTs that include larger sample sizes and subject subgroups are needed.

## Figures and Tables

**Table 1:** Detailed protocols for the included studies.

Study	Details of hormone administration		RCT study	Follow-up	Jadad score
	HRT group	Control group			
Guaschino et al.,2003 [6]	Equine conjugated estrogens 0.625 mg/day and dydrogesterone 5 mg/day in a continuous combined regimen; no ophthalmic treatment.	No hormonal or ophthalmic treatment.	Yes	1 year	3

Erdem et al., 2007 [14]	Conjugated estrogen 0.625 mg/day and medroxyprogesterone acetate 5 mg/day in a continuous combined regimen; no dry eye medication was given.	No hormone. Artificial tear products were allowed.	No	3 months	0

Affinito et al., 2003 [7]	Transdermal 17*β*-estradiol (E2), 50 *µ*g/day, and medroxyprogesterone acetate 10 mg/day for 12 days per cycle.	No hormonal or ophthalmic treatment.	Yes	3 months and 6 months	4

Taner et al., 2004 [15]	Estradiol plus medroxyprogesterone acetate (Premelle 5 mg), one tablet, qid.	No hormonal or ophthalmic treatment.	No	6 months	0

Scuderi et al., 2012 [13]	Phytoestrogen, one tablet, bid for 1 month; artificial tear products were allowed.	Placebo tablet. Artificial tear products were allowed.	Yes	1 month	6
Scuderi et al., 2012 [13] (reversed group)	Lacrisek tablet. Each Lacrisek tablet contained *α*-lipoic acid (100 mg), eicosapentaenoic acid (240 mg), and extract of fenugreek (200 mg), which contains diosgenin (1.3%), steroidal saponins (50%), and alkaloids; artificial tear products were allowed.				

HRT: hormone replacement therapy; RCT: randomly controlled trial.

**Table 2:** Demographic data of the included studies.

Study	Age		Menopause duration (year)		Number of participants (*n*)	
	HRT group	Control group	HRT group	Control group	HRT group	Control group
Guaschino et al., 2003 [6]	60.0 ± 5.4	59.8 ± 5.5	At least one year	At least one year	40	40
Erdem et al., 2007 [14]	50.2 ± 4.8	50.0 ± 4.6	3.2 ± 22	3.0 ± 1.6	20	5
Affinito et al., 2003 [7]	53.7 ± 5.9	51.9 ± 4.7	4.1 ± 2.7	3.4 ± 2.1	25	25
Taner et al., 2004 [15]	49.4 ± 5.5	52.1 ± 4.7	4.3 ± 4.4	5.7 ± 4.1	25	16
Scuderi et al., 2012 [13]	No data	No data	At least one year	At least one year	33	33

HRT: hormone replacement therapy.

**Table 3:** Schirmer test results of the included studies.

Study	Schirmer test under anesthesia	Schirmer test result (mm) before beginning the study		Schirmer test result (mm) after treatment		Calculated difference (mm) between values before beginning the study and after treatment		HRT effect
		HRT group	Control group	HRT group	Control group	HRT group	Control group	
Erdem et al., 2007 [14]	Yes	3.9 ± 1.1	4.1 ± 0.8	3.2 ± 0.9	4.1 ± 0.9	−0.7 ± 1.01	0 ± 0.85	Negative

Affinito et al., 2003a [7]	Yes	3.3 ± 1.5	3.2 ± 1.4	5.1 ± 1.3	3.4 ± 1.2	1.8 ± 1.41	0.2 ± 1.31	Positive

Guaschino et al., 2003 [6]	No	19.6 ± 8.1	17.8 ± 7.8	25.7 ± 7.9	17.4 ± 8.8	6.1 ± 8	−0.4 ± 8.35	Positive

Affinito et al., 2003b [7]	No	13.4 ± 2.1	13.5 ± 2.7	17.8 ± 2.4	14.0 ± 2.8	4.4 ± 2.26	0.5 ± 2.75	Positive

Taner et al., 2004 [15]	Not mentioned, but considered no	11.7 ± 0.9	12.8 ± 1.0	11.68 ± 1.0	12.7 ± 1.0	−0.02 ± 0.95	−0.1 ± 1	Negative

Scuderi et al., 2012a [13]	Not mentioned, but considered no	1.45 ± 1.32	1.36 ± 1.31	5.63 ± 3.16	1.45 ± 1.09	4.18 ± 2.75	0.09 ± 1.22	Positive

Scuderi et al., 2012b [13]	Not mentioned, but considered no	1.09 ± 1.18	2.00 ± 1.22	6.36 ± 2.84	1.18 ± 1.04	5.27 ± 2.47	−0.82 ± 1.14	Positive

HRT: hormone replacement therapy. Affinito 2003a and Affinito 2003b are the same study. The data for Schirmer tests with and without anesthetics were extracted and marked as Affinito 2003a and Affinito 2003b, respectively. Scuderi 2012a and Scuderi 2012b are the same study. The data for Schirmer tests from original grouping and reversed grouping are extracted and marked as Scuderi 2012a and Scuderi 2012b, respectively.

**Table 4:** Break-up time results of the included studies.

Study	Break-up time (s) before beginning of the study		Break-up time (s) after treatment		Calculated difference (s) between values after treatment and before beginning of the study		HRT effect
	HRT group	Control group	HRT group	Control group	HRT group	Control group	
Erdem et al., 2007 [14]	4.1 ± 0.9	4.2 ± 0.7	4.0 ± 0.5	4.2 ± 0.9	−0.1 ± 0.78	0.0 ± 0.82	Negative

Taner et al., 2004 [15]	12.0 ± 1.0	11.9 ± 0.9	12.2 ± 0.8	11.8 ± 1.0	0.2 ± 0.92	−0.1 ± 0.95	Negative

Scuderi et al., 2012a [13]	0.72 ± 0.761	0.72 ± 0.761	7.54 ± 2.09	0.90 ± 0.80	6.82 ± 1.83	0.18 ± 0.78	Positive

Scuderi et al., 2012b [13]	1.81 ± 1.35	1.72 ± 0.80	6.54 ± 2.91	0.60 ± 0.78	4.73 ± 2.52	−1.12 ± 0.79	Positive

HRT: hormone replacement therapy. Scuderi 2012a and Scuderi 2012b are the same study. The data of break-up time from original grouping and reversed grouping are extracted and marked as Scuderi 2012a and Scuderi 2012b, respectively.

**Table 5:** Results of the sensitivity analysis.

Excluded study	Schirmer test change with anesthetics			Schirmer test change without anesthetics			BUT change		
	Heterogeneity		Overall effect	Heterogeneity		Overall effect	Heterogeneity		Overall effect
	Chi^2^	*I* ^2^	*P* value	Chi^2^	*I* ^2^	*P* value	Chi^2^	*I* ^2^	*P* value
Erdem et al., 2007 [14]	Not applicable		<0.00001	/	/	/	16.52	99%	0.07
Affinito et al., 2003a [7]	Not applicable		0.11	/	/	/	/	/	/
Guaschino et al., 2003 [6]	/	/	/	129.95	98%	0.02	/	/	/
Affinito et al., 2003b [7]	/	/	/	130.74	98%	0.02	/	/	/
Taner et al., 2004 [15]	/	/	/	11.37	74%	<0.00001	259.69	99%	0.28
Scuderi et al., 2012a [13]	/	/	/	124.28	98%	0.03	171.07	99%	0.07
Scuderi et al., 2012b [13]	/	/	/	63.65	95%	0.02	96.47	98%	0.19

## References

[1] (2007). The definition and classification of dry eye disease: report of the definition and classification subcommittee of the International Dry Eye WorkShop. *The Ocular Surface*.

[2] Lemp M. A. (1995). Report of the national eye institute/industry workshop on clinical trials in dry eyes. *CLAO Journal*.

[3] McMonnies C. W., Ho A. (1987). Patient history in screening for dry eye conditions. *Journal of the American Optometric Association*.

[4] Doughty M. J., Fonn D., Richter D., Simpson T., Caffery B., Gordon K. (1997). A patient questionnaire approach to estimating the prevalence of dry eye symptoms in patients presenting to optometric practices across Canada. *Optometry & Vision Science*.

[5] Esmaeli B., Harvey J. T., Hewlett B. (2000). Immunohistochemical evidence for estrogen receptors in meibomian glands. *Ophthalmology*.

[6] Guaschino S., Grimaldi E., Sartore A. (2003). Visual function in menopause: the role of hormone replacement therapy. *Menopause*.

[7] Affinito P., Sardo A. D. S., Di Carlo C. (2003). Effects of hormone replacement therapy on ocular function in postmenopause. *Menopause*.

[8] Pelit A., Bagiş T., Kayaselçuk F., Dursun D., Akova Y., Aydin P. (2003). Tear function tests and conjunctival impression cytology before and after hormone replacement therapy in postmenopausal women. *European Journal of Ophthalmology*.

[9] Schaumberg D. A., Buring J. E., Sullivan D. A., Reza Dana M. (2001). Hormone replacement therapy and dry eye syndrome. *The Journal of the American Medical Association*.

[10] Moher D., Liberati A., Tetzlaff J. (2009). Preferred reporting items for systematic reviews and meta-analyses: the PRISMA statement. *Journal of Clinical Epidemiology*.

[11] Higgins J. P. T., Thompson S. G. (2002). Quantifying heterogeneity in a meta-analysis. *Statistics in Medicine*.

[12] Copenhagen R. Review Manager (RevMan) Version 5.

[13] Scuderi G., Contestabile M. T., Gagliano C., Iacovello D., Scuderi L., Avitabile T. (2012). Effects of phytoestrogen supplementation in postmenopausal women with dry eye syndrome: a randomized clinical trial. *Canadian Journal of Ophthalmology*.

[14] Erdem U., Ozdegirmenci O., Sobaci E., Sobaci G., Göktolga U., Dagli S. (2007). Dry eye in post-menopausal women using hormone replacement therapy. *Maturitas*.

[15] Taner P., Akarsu C., Atasoy P., Bayram M., Ergin A. (2004). The effects of hormone replacement therapy on ocular surface and tear function tests in postmenopausal women. *Ophthalmologica*.

[16] Higgins J. P. T., Green S. (2009). *Cochrane Handbook for Systematic Reviews of Interventions, Version 5.0.0*.

[17] Shapiro A., Merin S. (1979). Schirmer test and break-up time of tear film in normal subjects. *American Journal of Ophthalmology*.

[18] Clinch T. E., Benedetto D. A., Felberg N. T., Laibson P. R. (1983). Schirmer's test. A closer look. *Archives of Ophthalmology*.

[19] Serruya L. G., Nogueira D. C., Hida R. Y. (2009). Schirmer test performed with open and closed eyes: variations in normal individuals. *Arquivos Brasileiros de Oftalmologia*.

[20] Sullivan J. H., Crawford J. B., Whitcher J. P., Vaughan D., Asbury T., Riordan-Eva P. (1999). Schirmer test, clinical findings, dry eye syndrome, tears. *General Ophthalmology*.

[21] Lamberts D. W., Foster C. S., Perry H. D. (1979). Schirmer test after topical anesthesia and the tear meniscus height in normal eyes. *Archives of Ophthalmology*.

[22] Li N., Deng X.-G., He M.-F. (2012). Comparison of the Schirmer I test with and without topical anesthesia for diagnosing dry eye. *International Journal of Ophthalmology*.

[23] Gunduz K., Ozdemir O. (1994). Topical cyclosporin treatment of keratoconjunctivitis sicca in secondary Sjogren's syndrome. *Acta Ophthalmologica*.

[24] Behrens A., Doyle J. J., Stern L. (2006). Dysfunctional tear syndrome: a Delphi approach to treatment recommendations. *Cornea*.

